# Setting the radiological baseline: measuring natural radioactivity (^226^Ra, ^232^Th, ^40^K) in agricultural soils of Farta District, Ethiopia, and assessing potential health risks

**DOI:** 10.1093/jrr/rrag041

**Published:** 2026-06-04

**Authors:** Tadesse Abate Gobaw

**Affiliations:** Department of Physics, Debre Tabor University, Debre Tabor, Ethiopia

**Keywords:** natural radioactivity, baseline assessment, HPGe, AGDE, ELCR, Ethiopia

## Abstract

Tracking natural radioactivity, especially from naturally occurring radioactive material (NORM), matters for public health radiation concerns. We measured agricultural soils from Ethiopia’s Farta District using high-purity germanium gamma-ray spectrometry. There’s something people often miss: international guidelines like 1 mSv per year or 370 Bq/kg (for radium equivalent activity, Raeq) aren’t strict regulatory cutoffs, just broad screening tools. They help flag areas worth a closer look but don’t mean there’s a problem if background levels are naturally higher. Here’s what we found: 226Ra ranged from 12 to 342 Bq/kg, 232Th from 10 to 321 Bq/kg and 40 K from 65 to 671 Bq/kg. The averages are 144 ± 83, 141 ± 81 and 340 ± 182 Bq/kg for each, in order. Average Raeq was 370 ± 200 Bq/kg, which matches the usual international screening number, although some spots went above it. Factoring in an agricultural occupancy rate of 0.4 (because farmers spend more time outdoors), the average annual effective dose (AED) hit 0.41 mSv. If you assume a more general lifestyle with less outdoor exposure (occupancy 0.2), the AED drops to 0.20 mSv. So, you can really see how behavior shapes exposure. A number of samples went above the recommended marks for the internal hazard index (H_in) and annual gonadal dose equivalent. But again, these are just screening flags; they don’t mean there’s an immediate health threat. This work builds a strong baseline for the region. It gives future projects like environmental monitoring, food-chain tracking, radon measurements, or health studies a solid place to start, especially in a geologically unique area like Ethiopia’s Farta District.

## INTRODUCTION

Naturally occurring radionuclides are important for understanding radiation exposure and its health effects. Studying these elements improves our knowledge and safety measures related to radiation [1, 2]. Often overlooked, these materials can lead to significant radiation doses if not managed properly. Epidemiological studies have identified cancer risks from prolonged inhalation of naturally occurring radioactive materials [1–3]. These radionuclides take various forms due to radioactive decay, cosmic radiation and geological processes, emitting radiation as α, β and γ rays [4–9]. Key contributors to natural radiation include uranium (^238^U), thorium (^232^Th) and potassium (^40^K) [10–13]. Commonly found in rocks and granites, these radionuclides can be washed into the soil by rain, becoming important soil components. Significant radionuclides in soil, such as Uranium-238 and Thorium-232, have long half-lives, contributing to most of the annual dose from natural radiation [5, 14].

Their radiation, known as terrestrial background radiation, is often compared to reference levels set by organizations like International Commission on Radiological Protection (ICRP) and United Nation Scintific Committee on the Effect of Atomic Radiation (UNSCEAR). These groups recommend that radium equivalent concentration should not go over 370 Bq/kg, hazard indices should stay at or below 1 and the annual effective dose (AED) should not exceed 1 mSv/y for practices involving artificial sources or technologically enhanced naturally occurring radioactive materials [4–6, 15–20]. It is important to note that these limits are non-regulatory standards for unmodified natural background radiation. Instead, they act as screening guidelines to identify areas where radionuclide concentrations are higher than typical global levels and may need further investigation, especially when agricultural practices or land-use changes could mobilize or concentrate radioactive materials. Monitoring environmental radioactivity is crucial for public health, especially in rapidly developing areas, where excavation and industrial processing can increase natural radiation levels [21].

This research aims to address the lack of ecological radioactivity data for Farta, Ethiopia, by identifying key radionuclides and evaluating their hazards. With quick urbanization in Ethiopia, understanding naturally occurring and human-made radionuclide levels in soil is vital for public health and policy. Despite global studies measuring natural radioactivity [7–10, 22–25], data from Ethiopia remain limited, especially regarding its unique geology. Farta, located near the East African Rift Valley and Mount Guna, is particularly significant for studying soil radiation emissions [26–31].

This study provides a detailed baseline of natural radioactivity for a rift-influenced district in Ethiopia, which has limited data. The researchers combine precise high-purity germanium (HPGe) measurements with multivariate analyses to turn these measurements into useful information. In addition to reporting the levels of ^226^Ra, ^232^Th and ^40^K, they calculate standard dose and hazard indices that meet UNSCEAR guidelines. The study uses correlation, regression and clustering to uncover patterns in the radiological profile. Our goal is both practical and scientific. The study focuses on providing clear, geographically specific evidence to assist with surveillance, land-use decisions and identifying priority areas for further investigation, such as radon, food-chain transfer and ultimately epidemiology. In line with this aim, the study views the indices as model-based tools and does not make assumptions about health outcomes. In contrast, in the Farta District, you don’t find detectable ^137^Cs or ^60^Co in the soils. That fits with what we know: there aren’t any nuclear facilities nearby, and the area is far from global fallout sources. This absence backs up the idea that the high activity concentrations of ^226^Ra and ^232^Th here are natural, not the result of human activity.

## METHODOLOGY

### Description of site

The Farta district, which is located in the Amhara provincial state, is around 600 km away from the metropolis of Ethiopia, Addis Ababa. It is situated in the northwest plateau of Ethiopia between the coordinates 11^°^40^′^ to 12^°^2^′^ N North and 37^°^50^′^ to 38^°^18^′^E East. The entire land size of Farta district is 805.76 km^2^ [32]. It has been segregated into 32 administrative units or wards, which are known in Ethiopia as Kebeles. The district obtains an annual rainfall between 1097 and 1954 mm, with a long-term standard of 1248 mm [32]. The mean highest temperature is 21°C, experienced from February to May, and the lowest temperature is 9.6°C, from June to January, whereas the intermediate annual temperature is 15.5°C. The central statistics agency cast that the inhabitants of Farta district would acquire 234 143 in 2022, with the prevalence (80%) living in agrarian spots [32].

### Sample collection

Twenty-seven soil samples were collected from different locations in Farta, Ethiopia, at a depth of 5–9 cm, tracked using Global Positioning System (GPS). The samples were sieved through a 0.2 mm mesh and dried in an oven at 100°C for 24 hours. They were then placed in a 1 kg Marinelli beaker and sealed for at least 4 weeks to reach radioactive balance with radium and its decay products [9, 10]. The gamma-ray spectroscopy was used with a 50 mm diameter HPGe coaxial detector, which was shielded by an 8 cm-thick lead cylinder to reduce background radiation.

### Energy and efficiency calibrations

Gamma energy and efficiency calibrations used International Atomic Energy Agency (IAEA) reference sources (^60^Co, ^137^Cs, ^22^Na, ^241^Am, ^226^Ra) (Table 1). We calculated the HPGe detector’s efficiency using standard equations [10, 11]. We measured natural radioactivity with gamma-ray spectroscopy using an HPGe detector (0–1500 V) at 1332.5 keV (^60^Co), counting for 86 400 seconds to reduce error [10, 11, 33].

**Table 1:** γ–ray formal source for the energy calibration and efficiency calibration of HPGe detector in this investigation [34]

No¯.	Nuclide	γ-Energy	Activity (Bq·kg^−1^)	Abundance pγ(%)	Half-life	Intensity (%)	Source No¯.
		(keV)					
1	Americium-241	−26.34	35.6	36	432.2 years		RF 807
2	Caesium-137	661.6	35.8	85.1	30.2 years	85.1	RF 808
3	Cobalt-57	129	42.7	87	273 days	99.97	RF 809
4	Cobalt-60	1332.5	40	100	5.26 years	99.98	RF 810
5	Radium	351.9609.3	46.7	35.8,44.8	1620 years		RF 811

**Fig. 1:**
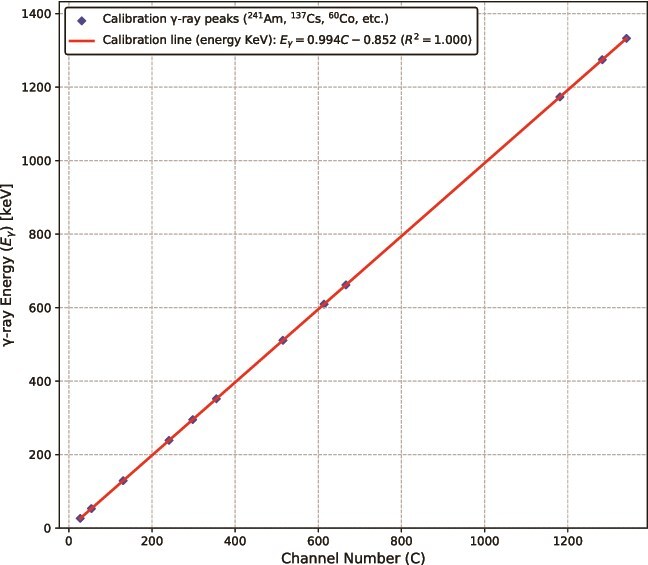
The energy emitted from the HPGe detector calibration curve.

**Fig. 2:**
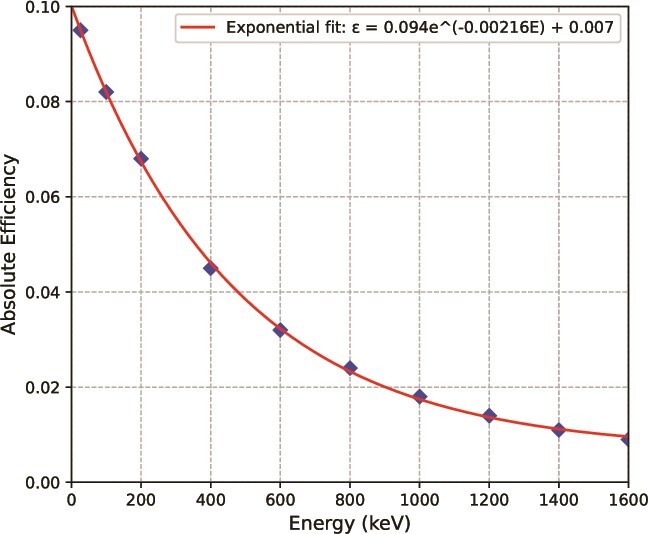
The HPGe transducer efficacy standardization curve.

Energy calibration coverage 26.3–1332.5 keV (0.0263, 0.0531, 0.1862, 0.6616, 1.1732, 1.2745, 1.3325 MeV) produced the linear fit *E*_y_ = 0.994*C* − 0.852, where *C* is the unitless channel number and was obtained with a perfect match (*R*^2^ = 1, Fig. 1). We conducted efficiency calibration following previous methods (Fig. 2 and Table 2) [7, 8]. Radionuclide analysis detected ^226^Ra (via ^214^Pb and ^214^Bi peaks), ^232^Th (via ^212^Pb, ^228^Ac, ^208^Tl) and ^40^K (1460.83 keV peak) [22, 34]. We measured samples for 24 hours, subtracting background counts using an empty container. Activity concentrations were calculated by subtracting background counts per Eq. (1) [11].

**Table 2:** Radionuclides with the energy of gamma ray [34]

Parent	Offspring	Energy (keV)	Abundance p*γ*(%)
232Th	212Bis	727.33	6.6
	212Pb	238.63	43.3
	228Ac	911.2	25.8
		968.97	15.8
	208Tl	510.77	22.6
		583.19	84.5
		860.56	12.4
		2614.53	99
226Ra	214Bi	609.31	46.1
		1120.28	15.1
		1764.49	15.4
	214Pb	295.22	19.3
		351.93	37.6
40K	40K	1460.83	11

### Calculation of radionuclide levels and derived indices

After sealing samples for at least four weeks, we assumed secular equilibrium between ^226^Ra/^232^Th and their short-lived progeny [11, 33]. Activities for ^226^Ra and ^232^Th were derived from progeny gamma lines (^226^Ra via ^214^Pb, ^214^Bi; ^232^Th via ^212^Pb, ^228^Ac, ^208^Tl) and for ^40^K from the 1460.8 keV line, following standard practice. Activity concentration A (Bq/kg) was computed as [9–11, 33]


(1) \begin{equation*} ACR\left( Bq\cdot{kg}^{-1}\right)=\frac{T}{G_{\gamma}\times{\eta}_{\gamma}\times{F}_g}{kg}^{-1} \end{equation*}

where T is the net photopeak count rate, *G*_γ_ is the emission probability, ${\eta}_{\gamma }$ is the full-energy peak efficiency and *F*_g_ is the sample mass (kg). Combined relative uncertainty was propagated from counting statistics, emission probabilities, efficiency calibration and mass measurement [7, 33].

The fate of the activity concentration (∆ACR) is premeditated with respect to element uncertainties, the count rate, the absolute transition probability of the specific gamma-ray, the mass of the sample and the efficiency of the detector as shadows [14];


(2) \begin{equation*} \Delta ACR\left( Bq\cdot{kg}^{-1}\right)= ACR\sqrt{\frac{\Delta T}{T}\times \frac{\Delta{G}_{\gamma }}{G_{\gamma }}\times \frac{\Delta{\eta}_{\gamma }}{\eta_{\gamma }}\frac{\Delta{F}_g}{F_g}}{kg}^{-1} \end{equation*}

where $\Delta T$ is the count rate fate; $\Delta{G}_{\gamma }$ is the emission prob ability fate; $\Delta{\eta}_{\gamma }$ is the detector efficiency fate and $\Delta{F}_g$ the mass fate.

#### Hazard indices due to ^226^Ra, ^232^Th and ^40^K values

The exposure to radiation springing from radionuclides of ^226^Ra, ^232^Th and ^40^K can be resolved in terms of some parameters, as given below.

##### Radium equivalent activity

The radium analogous activity (Ra_eq_), which is a single index, is used to depict the gamma output from distinct varieties of ^226^Ra, ^232^Th and ^40^K in the fabric. It was calculated from this formula [7–11].


(3) \begin{equation*} {Ra}_{eq}\left( Bq\cdot{kg}^{-1}\right)={ACR}_{Ra}+1.34\times{ACR}_{Th}+0.077\times{ACR}_K \end{equation*}

where ACR_Ra_, ACR_Th_ and ACR_K_ are the activity values of ^226^Ra, ^232^Th and ^40^K, respectively.

##### Absorbed dose rate

It is presumed that ionizing radiation from ^40^K, ^232^Th and ^226^Ra present in the soil will produce a strategy of radiation exposure. The radiation absorbed dose, ADR, is therefore used in radiation threat inspection to quantify the amount of radiation energy that may be absorbed per unit time on a potentially disclosed person [33]. The ADR, in nGy.h^−1^, in air at 1 m above foundation level was figured by involving modification factors of 0.462, 0.604 and 0.0417 for ^226^Ra, ^232^Th and ^40^K, respectively [1, 11] as follows;


(4) \begin{equation*} 0.462\times ADR={ACR}_{Ra}+0.604\times{ACR}_{Th}+0.0417\times{ACR}_K \end{equation*}

where ACR_Ra_, ACR_Th_ and ACR_K_ are the activity concentrations of ^226^Ra, ^232^Th and ^40^K, respectively.

##### Annual effective dose

An outdoor occupancy factor OF = 0.4 (≈9.6 hours/day) was adopted to reflect extended outdoor labor by local farmers [10, 11]. For transparency, AED values calculated with the general-population default OF = 0.2 are summarized in the Results and discussion [11], illustrating the sensitivity of model doses to time–activity assumptions. According to the evidence of [1], exterior and interior time habitation factors of 0.2 and 0.8, respectively, were instructed. These weights of the time characteristic insinuate that, on average, a person spends 4.8 hours alfresco and 19.2 hours in a building. In this study, we focus on outdoor radiation exposure for farmers. The outdoor occupancy factor (OF) used here does not reflect the general population; it is adjusted to account for the longer hours spent working in the fields by local rural Ethiopian agriculturalists. We adopted an outdoor OF of 0.4, which is equivalent to 9.6 hours per day. This value aligns with what was used by [33] in a similar rural situation in Nigeria. This change aims to provide a more accurate estimate of radiation exposure for farming communities. While the standard outdoor OF for the general population is 0.2, using this value would likely underestimate exposure for farming populations. The section Annual effective dose includes a discussion comparing sensitivities to show the difference in dose estimates if 0.2 were used. Employing this time factor, the A_ED_ affiliated with ADR was gauged utilizing equation (5).


(5) \begin{align*} AED &= ADR\times 8760\ h\times 0.4\times 0.7\ SvG. AED{y}^{-1}\times{10}^{-6} \nonumber \\&= ADR\times 2.4528\times{10}^{-3} \end{align*}

where ADR is the absorbed dose rate in air in nGy.h^−1^ and 8760 is the total hours in a year; 0.7 SvG.y^−1^ is the dose conversion factor from absorbed dose in air to the effective dose.

##### Internal hazard index

The inner hazard index (*H*_in_) of the gamma-ray specific activity concentrations of ^226^Ra, ^232^Th and ^40^K is computed by utilizing equation (6) given by [11, 34].


(6) \begin{equation*} {H}_{in}=\frac{ACR_{Ra}}{185}+\frac{ACR_{Th}}{259}+\frac{ACR_K}{4810}\le 1 \end{equation*}

##### External hazard index

The exterior imperilment index (*H*_ex_) is a depiction that calculates the exposure aspect and is a computation of the gamble of the crude gamma radiation due to the terrestrial radionuclides of ^226^Ra, ^232^Th and ^40^K. It can be figured employing equation (7) [9–11, 33, 34].


(7) \begin{equation*} {H}_{ex}=\frac{ACR_{Ra}}{370}+\frac{ACR_{Th}}{259}+\frac{ACR_K}{4810}\le 1 \end{equation*}

#### Level index for gamma

The gamma index is a radiological estimate meant to be employed in the screening of materials with attainable radiological health hindrance, notably when such materials are used for construction ambitions [33]. It was computed utilizing equation (8) [11, 33].


(8) \begin{equation*} {I}_{\gamma }=\frac{ACR_{Ra}}{150}+\frac{ACR_{Th}}{100}+\frac{ACR_K}{1500} \end{equation*}

The gamma index values of imply a radiologically safe soil, which corresponds to the upper limit of AED of 1 mSv [34].

#### Annual gonadal dose equivalent

To better evaluate the radiological influence of ^40^K, ^226^Ra and ^232^Th in the grange soil on the gonads, bone marrow and bone veneer cells, the annual gonadal equivalent dose (AGE) was calculated. As reported by the [1], these endogenous organs are most liable to radiation; therefore, they are deemed organs of crucial welfare. An accumulation in AGE simulates the bone marrow, foremost to the doom of the red blood cells, which are substituted by white blood cells. This circumstance can result in leukemia, a lethal blood cancer [33]. With the reckoned activity concentrations of ^40^K, ^226^Ra and ^232^Th, the AGE was figured utilizing the following correspondence.


(9) \begin{equation*} AGE=3.09\times{ACR}_{Ra}+4.18\times{ACR}_{Th}+0.314\times{ACR}_K \end{equation*}

#### Excess life time cancer risk

The excess lifetime cancer risk (ELCR) is employed in radiation peril inspection to forecast the probability of cancer development by an individual over a lifetime due to exposure to low-level radiation [4, 33]. Based upon the figured values of AED, the ELCR was calculated utilizing equation (10) [33].


(10) \begin{equation*} ELCR= AED\times ALD\times CRF \end{equation*}

where $AED$ is the annual effective dose, $ALD$ is the moderate lifetime period, presumed to be 70 years [33], and $CRF$ designates lethal cancer risk factor per sievert, taken to be 0.05 Sv^−1^ as retained in ICRP-103 publication [19].

#### Exposure rate

The rate of gamma–ray exposure in the ambiance, reckoned at a unique meter beyond a viscous block that is immensely inflated, employing ^238^U (^226^Ra), ^232^Th series and ^40^K that were consistently disseminated within the fabric, comprises as [35]


(11) \begin{equation*} X\left(\mu R.{h}^{-1}\right)=1.9\times{ACR}_{Ra}+2.82\times{ACR}_{Th}+1.97\times{ACR}_K \end{equation*}

where *X* is portrayed as the divulgence rate $\left(\mu R.{h}^{-1}\right)$. Herein, the levels of activity are frequently offered in pCi/g. For a single radionuclide in the nuclear decay cascade, the moderate weights of the gamma–ray powers are characterized by the everlastings exemplified on the starboard flank of equation (11).

## RESULTS AND DISCUSSION

### Activity concentrations in soil

**Fig. 3:**
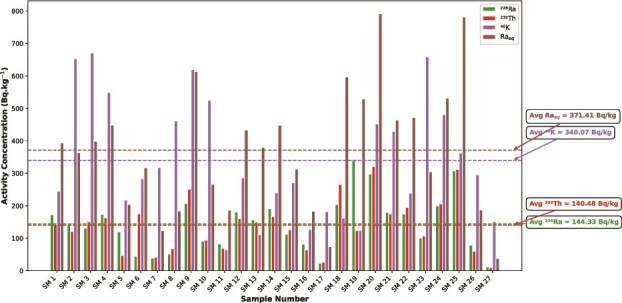
Baseline radioactivity levels in agricultural soils of the study site.

Table 3 sums up the activity concentrations of ^226^Ra, ^232^Th and ^40^K in 27 surface soil samples from the Farta District, with Fig. 3 showing the spread. The numbers jump around quite a bit depending on where the sample came from ^226^Ra ranges from 12 to 342 Bq/kg, ^232^Th from 10 to 321 Bq/kg and ^40^K from 65 to 671 Bq/kg. The figured averages are 144 ± 83 Bq/kg for ^226^Ra, 141 ± 81 Bq/kg for ^232^Th and 340 ± 182 Bq/kg for ^40^K (see Table 4). The standard deviations here have significant values that are about half or more of the mean for each radionuclide. That kind of spread points to a lot of spatial heterogeneity in how these radionuclides are distributed, and it probably ties back to differences in parent material, varying soil properties and how people manage the land throughout the district.

**Table 3:** Activity concentrations of ^226^Ra, ^232^Th, ^40^K (Bq.kg^−1^), activity equivalent of radium Ra_eq_, Bq.kg^−1^) and absorbed dose rate (ADR, nGy.h^−1^) measured in farmland soils

S. code	Latitude	Longitude	^226^Ra	^232^Th	^40^K	Ra_eq_	ADR
SM1	11.726825	38.133781	172 ± 52	142 ± 47	245 ± 74	394 ± 80.0	175.5 ± 35.2
SM2	12.024534	38.227597	140 ± 42	121 ± 36	653 ± 196	363 ± 68.5	165.0 ± 30.4
SM3	11.911521	37.57244	131 ± 39	151 ± 45	671 ± 201	399 ± 77.3	179.7 ± 33.9
SM4	11.842627	38.245754	173 ± 52	163 ± 49	549 ± 165	448 ± 88.0	201.3 ± 38.7
SM5	11.613915	37.721329	120 ± 36	47 ± 14	218 ± 65	204 ± 41.6	92.9 ± 18.9
SM6	11.613902	37.594762	45 ± 14	175 ± 52	283 ± 85	317 ± 76.6	138.3 ± 32.5
SM7	11.563312	38.655363	39 ± 12	42 ± 13	318 ± 95	124 ± 22.7	56.7 ± 10.2
SM8	11.980853	38.675458	51 ± 15	68 ± 20	462 ± 139	184 ± 34.6	83.9 ± 15.3
SM9	11.843896	38.486777	207 ± 62	251 ± 75	620 ± 186	614 ± 125.1	273.1 ± 54.3
SM10	11.899161	37.882237	91 ± 27	94 ± 28	525 ± 158	266 ± 50.2	120.7 ± 22.2
SM11	11.543936	37.633907	83 ± 25	69 ± 21	65 ± 20	187 ± 38.7	82.7 ± 17.0
SM12	12.034452	38.33778	181 ± 54	161 ± 48	286 ± 86	433 ± 88.1	192.8 ± 38.6
SM13	11.963423	38.044883	157 ± 47	150 ± 45	111 ± 33	380 ± 79.8	167.8 ± 34.9
SM14	11.643016	37.663146	191 ± 57	167 ± 50	240 ± 72	448 ± 91.9	199.1 ± 40.3
SM15	11.627249	38.110912	112 ± 34	126 ± 38	271 ± 81.	313 ± 64.0	139.2 ± 27.8
SM16	11.628065	37.557966	82 ± 25	64 ± 19	127 ± 38	183 ± 37.0	81.8 ± 16.3
SM17	11.690502	38.607884	23 ± 7	26 ± 8	182 ± 55	74 ± 13.8	33.9 ± 6.1
SM18	11.804442	37.827236	204 ± 61	266 ± 80	162 ± 49	597 ± 129.6	261.7 ± 55.9
SM19	11.756486	38.311727	342 ± 103	124 ± 37	125 ± 38	529 ± 115.6	238.1 ± 52.5
SM20	11.683778	37.890753	298 ± 89	321 ± 96	452 ± 136	792 ± 164.5	350.4 ± 71.6
SM21	11.849444	38.140782	180 ± 54	175 ± 53	429 ± 129	463 ± 93.0	206.8 ± 40.7
SM22	11.605376	38.172752	175 ± 53	195 ± 59	239 ± 72	472 ± 98.9	208.6 ± 43.0
SM23	11.684251	37.738525	101 ± 30	107 ± 32	659 ± 198	305 ± 57.1	138.8 ± 25.3
SM24	11.722599	38.680202	200 ± 60	206 ± 62	481 ± 144	532 ± 107.4	236.9 ± 46.9
SM25	11.768951	38.446859	308 ± 92	312 ± 94	362 ± 109	782 ± 162.9	345.8 ± 71.0
SM26	11.939	38.644099	79 ± 24	60 ± 18	295 ± 89	188 ± 35.7	85.0 ± 15.9
SM27	11.636471	38.590493	12 ± 4	10 ± 3	152 ± 46	38 ± 6.6	17.9 ± 3.1

S. = sample; the ± values = actual measurement uncertainties (SDs).

**Table 4:** Summary statistics for ^226^Ra, ^232^Th, ^40^K and Ra_eq_ concentrations and ADR in the analyzed soil

Description	^226^Ra	^232^Th	^40^K	Ra_eq_	ADR
Minimum	12 ± 4	10 ± 3	65 ± 20	38.0 ± 6.6	17.92
Maximum	340 ± 103	321 ± 96	671 ± 201	792 ± 164.5	350.41
Mean	144	141	340	370	165.7
SD	83	81	182	195	81.1
Skewness	0.54	0.52	0.43	0.37	0.35
Kurtosis	−0.15	−0.30	−1.02	−0.33	−0.35
*t*-statistics	8.82	8.85	9.55	9.89	10.01
*P*–value	0.0001	0.0002	0.0001	0.0003	0.0002
95% confidence interval	144.33 ± 33.0	140.48 ± 32.01	340.07 ± 71.84	371.41 ± 77.21	165.71 ± 32.22

SD, standard deviation.

Compared to the global averages UNSCEAR reports (35 Bq/kg for ^226^Ra, 30 Bq/kg for ^232^Th and 400 Bq/kg for ^40^K) [36], soils in Farta District stand out. The mean concentrations of ^226^Ra and ^232^Th are about four and five times higher, while ^40^K is roughly 15% lower than the global mean. This pattern elevated ^226^Ra and ^232^Th, with ^40^K hovering just below or around average, shows up in other studies from Ethiopia’s rift-influenced highlands as well. It points to the volcanic rocks beneath these soils, which tend to carry more uranium- and thorium-rich minerals. The lower ^40^K suggests either a scarcity of potassium-rich rocks in the area or that weathering and leaching have stripped potassium away over time. The results show that the average concentrations of ^226^Ra and ^232^Th in this study outstrip the global average values by 381.1% and 301.37%, respectively. The more towering average activity concentrations of ^226^Ra and ^232^Th in Farta District soils likened to global values may result from:

- Regional soil composition, Parent rock types and geological formation processes;
- The region’s unique geology, including uranium/thorium-rich parent rocks (e.g. granite or monazite sands);
- It may be due to possible use of phosphate fertilizers, which often contain elevated radionuclides [25, 37];
- Soil properties (e.g. clay content) that enhance radionuclide retention.

In contrast, the lower ^40^K levels could reflect the presence of reduced potassium-bearing minerals in the local geology. However, their dominance over ^40^K and ^40^K suggests endowments from potassium-rich compost. Notably, ^40^K exhibits higher activity concentrations than ^226^Ra and ^232^Th across all analyzed samples, confirming its dominance as the most abundant terrestrial radionuclide in the studied soils. This elevated ^40^K level could also be attributed to the use of potassium-rich fertilizers in these agrarian zones. All in all, the results portray that samples taken near swamps and from areas where rice and onions are grown repeatedly every year have higher concentrations of natural radionuclides than samples taken from dry areas and areas where crops are grown only once a year. This may be due to the use of highly phosphorus-rich fertilizers because phosphate fertilizers can add up to 1000 Bq.kg^−1^ of U–238 (Ra–226) to soil [38], and so, permanent use boosts soil radioactivity by 10–25% [36]. Once again, potash fertilizers contribute ∼300–600 Bq.kg^−1^ of K–40 as reported by Szabo *et al*. [39]. Because fertilizers are used to increase the fertility of the soil by increasing the concentrations of phosphorus and potassium, as well as high anthropogenic sources, such as ^137^Cs and ^60^Co.

The activity concentrations of ^226^Ra, ^232^Th and ^40^K in the analyzed soil samples demonstrated substantial variability, ranging from 12 ± 4 to 342 ± 103 Bq.kg^−1^, 10 ± 3 to 321 ± 96 Bq.kg^−1^ and 65 ± 20 to 671 ± 201 Bq.kg^−1^, respectively. The corresponding mean ± standard deviation values were 144 ± 83 Bq·kg^−1^ for ^226^Ra, 141 ± 82 Bq·kg^−1^ for ^232^Th and 340 ± 182 Bq·kg^−1^ for ^40^K (see Table 4 and Fig. 3). All three radionuclides exhibited right-skewed distributions (skewness = 0.37–0.54), indicating a propensity for elevated concentrations in a subset of samples. Additionally, the distributions were platykurtic (kurtosis = −1.02 to −0.15), implying flatter-than-normal curves characteristic of environmental data influenced by spatial or geochemical outliers. The pronounced standard deviations, amounting to ~50–60% of the respective means, further underscore the marked spatial heterogeneity in radionuclide levels across the study area. Statistical analysis revealed highly significant differences from global reference values, with *t*-statistics ranging from 8.82 to 9.89 (*P*-value ≤ 0.001), confirming the elevated and non-random nature of the measured concentrations.

**Fig. 4:**
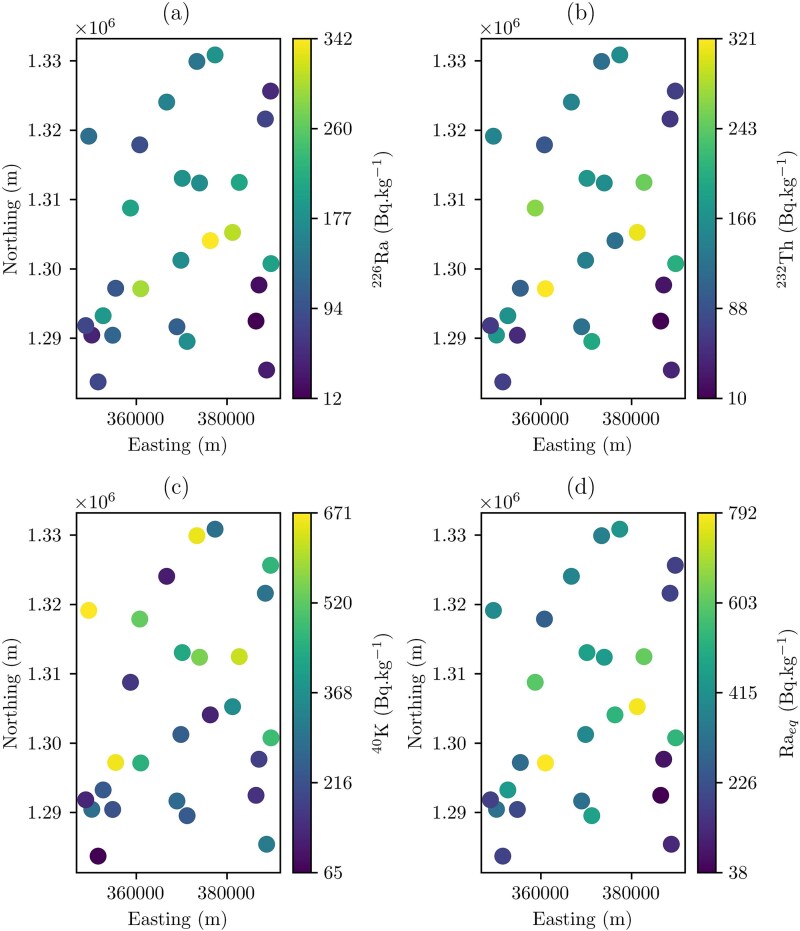
Machine learning–interpolated spatial distribution of radionuclide concentration (a) ^226^Ra, (b) ^232^Th, (c) ^40^K and (d) Ra_eq_ in Farrta District, Ethiopia.

Figure 4 depicts a geospatial analysis of three naturally occurring radionuclides (^226^Ra, ^232^Th and ^40^K) and the radium equivalent activity (Ra_eq_) over the Farta District. Each subplot (Fig. 4a–d) maps isotopic concentrations (Bq·kg^−1^) as scatter points, where color intensity (colormap: lighter hues indicate higher values, darker hues lower values) reflects activity levels ^226^Ra (12–342 Bq·kg^−1^), ^232^Th (10–321 Bq·kg^−1^) and ^40^K (65–671 Bq·kg^−1^). The Ra_eq_ map (Fig. 4d) confirms that the radium equivalent hotspots coincide geographically with elevated ^226^Ra and ^232^Th concentrations, reinforcing the dominant contribution of these two radionuclides to the overall radiological hazard. The pronounced color gradients reveal spatial heterogeneity, with localized hotspots (e.g. ^226^Ra *>* 300 Bq·kg^−1^ in eastern areas) and coldspots (e.g. ^40^K *<* 100 Bq·kg^−1^ in scattered regions). These variations likely arise from geological and environmental factors may be due to: (i) lithological control, where high ^226^Ra and ^232^Th correlate with uranium- and thorium-rich bedrock or weathered products; (ii) sedimentary processes, redistributing radionuclides along hydraulic gradients; (iii) agricultural influence, as ^40^K concentrations may reflect potassium fertilizer use or soil type differences; and (iv) anthropogenic contamination, such as mining or improper waste disposal elevating localized radioactivity. The spatial clustering of high values suggests non-random distributions, possibly tied to fault zones, mineralization or land use. By visualizing these patterns, the figure aids in identifying risk zones and guiding further investigations into natural versus anthropogenic drivers of radioactivity.

Ra_eq_ values ranged from 38 to 791.8 Bq/kg, with an average of 370 ± 200 Bq/kg. This places the district at the threshold of the 370 Bq/kg screening level. The exceedances were confined to certain areas rather than widespread. This highlights the need for management tailored to specific sites instead of general conclusions. Considering the caution in screening indices and uncertainties in occupancy and pathways, these results identify priority areas for targeted measurement and mitigation, without implying definitive health risks.

**Fig. 5:**
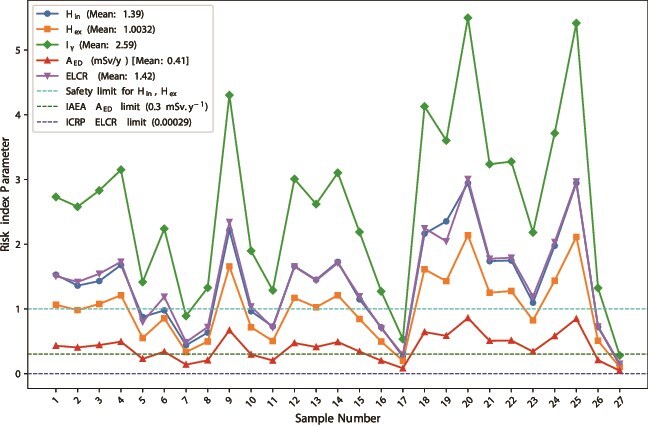
Radiological impact assessment based on dose and hazard indices in the sampling area.

The measured activity concentrations of ^226^Ra (144 Bq.kg^−1^), ^232^Th (141 Bq.kg^−1^) and ^40^K in Farta District soils align closely with other Ethiopian studies (e.g. 105.34–398 Bq.kg^−1^ for ^226^Ra; 40.7–235 Bq.kg^−1^ for ^232^Th; 161.6–562.8 Bq.kg^−1^ for ^40^K), confirming regional geological homogeneity in uranium/thorium enrichment (visit Fig.  5 and Table 5). Notably, Farta’s ^226^Ra and ^232^Th values exceed global averages (35 Bq.kg^−1^ and 30 Bq.kg^−1^, respectively; [36]) by factors of 4–5, reflecting Ethiopia’s geogenic anomaly linked to the East African Rift’s volcanic lithology. However, they remain lower than extremely high-background areas (e.g. 398 Bq.kg^−1226^Ra in Ethiopia [7] or 151.73 Bq.kg^−1232^Th in Nigeria [33]). Farta’s ^40^K is consistent with Ethiopian ranges (161.63–562.8 Bq.kg^−1^) but lower than potassium-rich regions like Tunisia [33], likely due to differences in soil fertility and agricultural inputs. Critically, Farta’s ratios of ^226^Ra/^232^Th (∼1.03) and ^226^Ra/^40^K (∼0.42) suggest a balanced uranium–thorium decay series dominance, distinct from regions with skewed ratios, in India’s ^226^Ra/^40^K = 0.13 [15]. These findings underscore the necessity for localized radiation risk assessments, as Ethiopia’s rift-related geology drives elevated but variable radionuclide distributions compared to global norms.

**Table 5:** Comparison of mean activity concentrations (Bq kg^−1^) ^226^Ra, ^232^Th and ^40^K in soil samples from Farta District with published values from different regions of Ethiopia and other geologically distinct regions abroad

Origin of study	^226^Ra	^232^Th	^40^K	Reference
Ethiopia	265.7	40.7	161.6	[4]
Ethiopia	398	235	562.8	[7]
	28.3	21.2	235	[7]
Ethiopia	64	70	330	[8]
Ethiopia	105.3	123.1	371.4	[10]
India	58.0	83.9	455.2	[15]
Iraq	26.2	18.7	307.6	[21]
	20	15	283	[22]
Bangladesh	27	37	499	[23]
Tunisia	21.8	26.5	559.2	[27]
	26.6	24.5	661.3	[27]
Nigeria	3.35–151.7	0.72–154.9	BDL–468.2	[33]
	BDL–241.7	BDL–150.2	BDL–607.1	[33]
Ethiopia	144	141	340	Our result
UNSCEAR	35	30	412	[35]

BDL, below detectable level.

Relative to UNSCEAR [36] global references, which are 35 Bq/kg for ^226^Ra, 30 Bq/kg for ^232^Th and 400 Bq/kg for ^40^K, Farta soils show higher levels of ^226^Ra and ^232^Th and somewhat lower levels of ^40^K. This pattern matches the U–Th enrichment found in rift-related Ethiopian rocks and parts of East Africa. The average outdoor ADR is ~165.7 nGy/h, which is higher than the global outdoor average of around 59 nGy/h but still falls within the ranges seen in areas with natural elevation worldwide. This information supports a targeted monitoring approach that focuses on hotspots instead of applying uniform restrictions across all areas.

This study is limited by the absence of measurements for anthropogenic radionuclides such as ^137^Cs and ^60^Co, which would be necessary to definitively exclude the influence of nuclear fallout or other human activities on the observed radiological profile. Cesium-137, in particular, serves as a sensitive tracer for contamination from nuclear weapons testing and reactor accidents, with detectable levels persisting in soils decades after deposition due to its 30.2-year half-life and strong affinity for clay minerals. The absence of such data means that while the elevated ^226^Ra and ^232^Th concentrations observed in Farta soils are consistent with the region’s volcanic geology, a small contribution from anthropogenic sources cannot be rigorously ruled out. Future monitoring efforts in the district should therefore include gamma spectroscopy analysis for ^137^Cs and ^60^Co to establish a complete radiological baseline that captures both natural and potential anthropogenic components.

### Risk assessments due to natural radioactivity

Radiological impact assessment is how it functions in reckoning and gauging the probable health consequences that would result from radiation exposure. The stock of radiation dose can be estimated by applying diverse dosimetry scenarios, environmental measurements and computer simulations [4]. Formerly, the radiation doses were examined, and a health risk estimation was accomplished to consider the conceivable unfavorable health-related impact. Epidemiologic investigations, creature examinations and further technological particulars are employed to appraise the perils associated with dissimilar radiation doses. The reckoned radiation perils paralleled designated radiation security benchmarks and approaches. These standards furnish reference levels or limits for radiation exposure to protect someone and the populace from disproportionate radiation doses [4].

Can attend in Table 4, the computed gamma absorbed dose rate (ADR) goes from 17.9 to 350.4 nGy.h^−1^, with a prevailing average of 165.7 nGy.h^−1^, which is below the guided value [17]. According to UNSCEAR (2000), 65% of the global average outdoor dose rate of 59 nGy h^−1^ is contributed by terrestrial gamma radiation. As shown in Table 6 and Fig.  7, the calculated AED ranges from 0.04 to 0.86 mSv/y, with an average value of 0.41 mSv/y based on an agricultural occupancy factor of 0.4. This average is about six times higher than the global average of 0.07 mSv/y reported by UNSCEAR [17, 36]. For context, the ICRP public dose limit of 1.0 mSv/y applies to practices involving artificial radiation sources or controlled NORM industries, not to unmodified natural background [19]. However, using this value as a conservative screening benchmark, all measured AED values are below the 1.0 mSv/y limit. This shows that even with prolonged outdoor exposure (9.6 hours per day), the estimated radiation doses stay within ranges typically deemed acceptable for comparison with controlled radiation practices. The investigation site offered a more elevated radiation inconsistency. Such radiologic segments can integrate along with meals and subsequently pass up the food cycle to greenery, creatures and individuals. On the contrary, the outward hazard indicator of innately materializing reacting radiation was in the range of 0.10 and 2.14, with a moderate value of 1.003. The interior disclosing to ^222^Rn (daughter of radium) and its offspring is exploited by the interior indicator, which is within 0.14 and 2.94, with a standard of 1.39. The outward peril index is almost equivalent to the globally quoted value, whereas the internal hazard index is higher than the recommended limit. In addition, the gamma representative index ranges from 0.28 to 5.50, with a mean value of 2.59, which is 2.59 times higher than the recommended safety limit.

**Table 6:** Overview of radiological risk indicator parameters assessments conducted on measured soil

S. code	H_in_	H_ex_	G_γ_	AGDE (Sv·y^−1^)	AED (mSv·y^−1^)	ELCR (×10^−2^)	X (μR·h^−1^)
SM1	1.53	1.06	2.73	1201.97	0.43	1.51	775.51
SM2	1.36	0.98	2.58	1143.42	0.4	0.4	735.86
SM3	1.43	1.08	2.83	1246.66	0.44	1.54	806.91
SM4	1.68	1.21	3.15	1388.3	0.49	1.73	896.51
SM5	0.88	0.55	1.42	635.71	0.23	0.8	403.49
SM6	0.98	0.86	2.24	959.41	0.34	1.19	654.75
SM7	0.44	0.33	0.89	395.92	0.14	0.49	255.19
SM8	0.63	0.5	1.33	586.9	0.21	0.72	379.67
SM9	2.22	1.66	4.3	1883.49	0.67	2.34	1223.26
SM10	0.96	0.72	1.9	838.96	0.3	1.04	541.4
SM11	0.73	0.5	1.29	565.3	0.2	0.71	365.08
SM12	1.66	1.17	3.01	1322.07	0.47	166	854.26
SM13	1.45	1.03	2.62	1146.98	0.41	1.44	743.17
SM14	1.73	1.21	3.1	1363.61	0.49	1.71	881.12
SM15	1.15	0.85	2.19	957.85	0.34	1.19	621.51
SM16	0.72	0.5	1.27	560.78	0.2	0.7	361.3
SM17	0.26	0.2	0.53	236.9	0.08	0.29	152.87
SM18	2.16	1.61	4.13	1793.11	0.64	2.25	1169.63
SM19	2.35	1.43	3.6	1614.35	0.58	2.04	1024.1
SM20	2.94	2.14	5.5	2404.53	0.86	3.01	1560.46
SM21	1.74	1.25	3.24	1422.41	0.51	1.77	920.01
SM22	1.75	1.28	3.28	1430.9	0.51	1.79	929.48
SM23	1.1	0.82	2.18	966.28	0.34	1.19	623.46
SM24	1.98	1.44	3.71	1630.11	0.58	2.03	1055.68
SM25	2.94	2.11	5.41	2369.55	0.85	2.97	1536.35
SM26	0.72	0.51	1.32	587.54	0.21	0.73	377.42
SM27	0.14	0.1	0.28	126.61	0.04	0.15	80.94
Min	0.14	0.1	0.28	126.61	0.04	0.15	80.94
Max	2.94	2.14	5.5	2404.53	0.86	3.01	1560.46
SD	0.74	0.53	1.35	588.51	0.21	0.74	374.56
Average	1.39	1.003	2.59	1139.99	0.41	1.42	737.39

Keywords: Min = ‘minimum’, Max = ‘maximum’ and S. = ‘sample’, SD = standard deviation.

The ELCR based on AED estimates falls between 0.15 × 10^−2^ and 3.01 × 10^−2^, with an average of 1.42 × 10^−2^ (see Table 6*).* That average is about five times higher than the recommended screening level of 0.29 × 10^−3^ [7, 11, 20, 33, 34]. But it’s important to be clear about these numbers. As already mentioned in Introduction and Risk assessments due to natural radioactivity, the 1 mSv/y public dose limit set by the ICRP only covers artificial radiation sources, not regular natural background radiation. So, what we’re doing here is a screening check unstrict regulatory or legal compliance test. Even the highest AED recorded, 0.86 mSv/y, still stays under the 1 mSv/y reference level when used for screening. It’s also worth remembering that ELCR doesn’t actually count cancer cases directly. Instead, it’s a theoretical projection based on several cautious assumptions: a linear no-threshold model for dose–response, continuous exposure over 70 years and specific occupancy and conversion values (see the Annual effective dose section). These estimates are useful for initial evaluation, but they shouldn’t be confused with actual risk predictions. These models don’t consider things like dose-rate effects, how cells repair themselves or differences between individuals. So, even though the higher ELCR values highlight the Farta District as an area that needs attention, they don’t mean people there will actually get cancer at those rates. To critically understand any health impact, researchers need to conduct local epidemiological studies and look for actual connections between background radiation and health in this community. The value suggested by the ICRP for occupational exposures typically spans between 0.05% and 5% per sievert (Sv) of effective dose [19]. Correspondingly, the UNSCEAR furnished an authority value of 0.2% to 4% per Sv of effective dose [36]. By the upshot, out of 1000 individuals, up to 50 will have a likelihood of acquiring cancer as a result of long-lasting exposure to radiation. According to these reckoned significances, it may be inferred that the radiation influence in the investigation site is substantial. Furthermore, the annual gonadal equivalent dose (AGDE) values in the studied soils ranged from 126.6 to 2404.5, with the prevailing average of 1140 mSv.y^−1^. As can be seen, the AGDE average value is far more generous than the instructed boundary of 300 mSv.y^−1^ [1], signifying that around farming areas, the gonads, bone marrow and the bone surface cells of the farmers are vastly at risk. As well, it was uncovered that the utmost value of *X* in instance SM20 was 1560.5 *μ*R.h^−1^ and the smallest value in instance SM27 was 80.9 *μ*R.h^−1^, with a despicable value of 737.4 ± 374.5 *μ*R.h^−1^. The treasure of divulgence *X*, is remarkably more elevated than the finding by Ibrahim *et al.* [35]. These measurements were calculated using equation (11), which takes into account the specific activity concentrations of radionuclides like ^226^Ra, ^232^Th and ^40^K present in the soil samples. The calculated exposure rates are remarkably variable, with values ranging from as low as 80.9 μR.h^−1^ for sample SM27 to a striking high of 1560.5 μR.h^−1^ for sample SM20. The overall mean exposure rate across all samples is ~737.4 ± 374.5 μR.h^−1^, as detailed in Table 6. This average is particularly noteworthy when compared to previous studies. For instance, research by Ibrahim *et al*. [35] reported a much lower mean exposure rate of just 134 μR.h^−1^ for similar soils in Iraq, making the values observed in the Farta District over five times higher. Such a stark difference strongly implies that the soils in Farta District contain considerably elevated concentrations of ^226^Ra and ^232^Th, which are the primary contributors to external gamma radiation in terrestrial environments.

The variability in exposure rates in this region is not just significant; it is dramatic, with almost two orders of magnitude separating the lowest and highest measurements. This pronounced fluctuation reflects the highly heterogeneous distribution of natural radionuclides within the area, echoing earlier findings from radionuclide concentration analysis. Notably, the samples exhibiting the most elevated exposure rates (SM20, SM25, SM9 and SM18) are consistent with the locations identified as hotspots in both the spatial distribution maps (Fig. 4) and the radiological hazard index plots (Fig.  5). This reinforces the reliability of the dataset and underscores the spatial clustering of radiological hazards in the region.

To provide further context, converting the mean gamma-ray exposure rate to an ADR (using the standard factor where 1 μR.h^−1^ equates to ~8.7 nGy.h^−1^), the average exposure rate of 737.4 μR.h^−1^ translates to ~6415 nGy.h^−1^. This value aligns closely with ADRs directly calculated from the radionuclide activity concentrations (mean ADR = 165.7 nGy.h^−1^), when accounting for necessary unit conversions and background radiation levels. This agreement between different calculation methods serves to validate the robustness of the results.

The implications of these findings are significant. Certain locations within the Farta District exhibit notably high gamma-ray exposure rates, suggesting that external gamma radiation is a major contributor to the overall radiological burden in these environments. This is particularly relevant for land-use planning and public health, especially in areas frequented by people for agriculture or other outdoor activities. Elevated exposure rates may warrant additional precautions or restrictions on land use to mitigate potential health risks. However, it is crucial to recognize that these calculations are preliminary estimates, intended to highlight areas that may require more detailed investigation. They do not provide a comprehensive risk assessment or definitive conclusions about health impacts.

To move beyond these initial findings, it is advisable to conduct follow-up field studies using portable gamma survey meters to directly measure on-site exposure rates. Such ground truthing would help confirm the presence and extent of radiological hotspots, providing a clearer basis for risk assessment and management decisions. In summary, while the current data suggest the need for vigilance and further study, it also offers a valuable first step in identifying and prioritizing areas for more focused radiological monitoring and potential remediation.

**Fig. 6:**
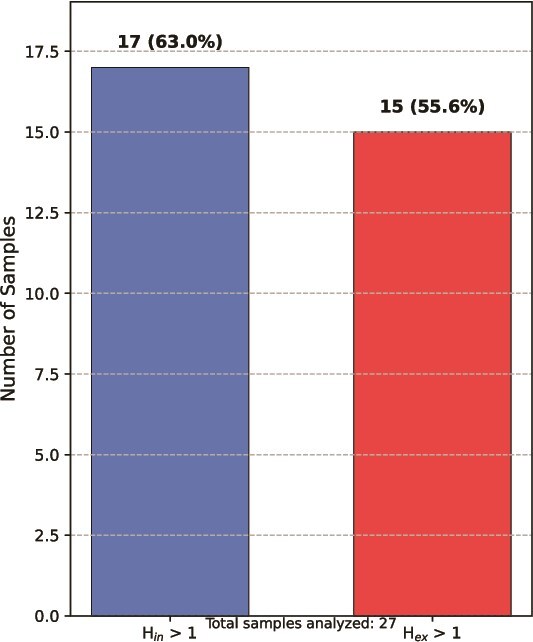
Number of soil samples (out of 27) exceeding the internal hazard index (*H*_in_ > 1) and external hazard index (*H*_ex_ > 1) limits. The internal hazard index exceeds the recommended value in 17 samples, which is 63.0%, while the external hazard index exceeds in 15 samples, or 55.6%. Percentages are calculated based on the total number of samples. These indices serve as screening tools. Exceeding the limits indicates potential concern that needs further investigation, but it does not mean there is a definite health risk.

Model-based indicators (ELCR, AGDE, *H*_in_, *H*_ex_) surpassed reference levels at several sites. This shows areas of potential concern that require specific follow-up, such as radon surveys, inhalable particulates and crop ingestion. These indices come from standard conversion factors and do not provide proof of disease burden. In addition, Fig. 6 presents a bar chart that compares samples exceeding hazard index limits: *H*_in_ > 1 (blue, internal hazard) and *H*_ex_ > 1 (red, external hazard) [1, 36]. Out of 27 samples, 17 (62.96%) exceed the internal limit, and 15 (55.56%) surpass the external limit. The chart features labeled axes, a title, percentage labels and a grid for clarity. A note at the bottom shows the total number of samples. The design is clean and avoids unnecessary borders. It emphasizes that most samples go beyond both thresholds, with internal hazards being slightly more frequent.

While all calculated AEDs in Table 6 are below the 1 mSv/y public dose limit (ICRP 103) [3], we conducted a sensitivity analysis to assess the impact of occupancy factors. Using the standard 0.2 factor for the general population instead of the agricultural 0.4 factor lowered A_ED_ values by 48 to 52% across all sites. For example, SM20 decreased from 0.86 to 0.43 mSv/y. Even with the higher 0.4 factor, the maximum observed AED at SM20 was still 0.86 mSv/y, remaining below regulatory limits. This suggests that radiological risks are acceptable for both farmers and the general population. However, the 0.4 factor more accurately reflects actual exposure for agricultural workers who spend about 9.6 hours a day outdoors, according to the same environmental study by Ugbede *et al*. [33]. This analysis shows that while absolute doses change with occupancy time, all measured values meet international standards.

**Fig. 7:**
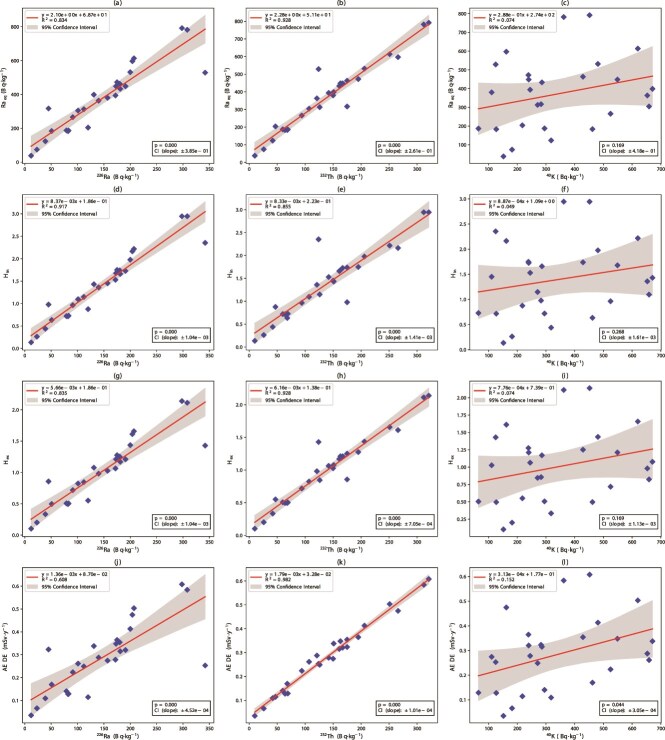
Linear regression analysis between individual radionuclide activity concentrations (^226^Ra, ^232^Th, ^40^K) and four key radiological indices: radium equivalent activity (Ra_eq_, panels a–c), internal hazard index (*H*_in_, panels d–f), external hazard index (*H*_ex_, panels g–i) and annual effective dose (AED, panels j–l). Each panel shows the least-squares best-fit line, 95% confidence band (shaded), regression equation, coefficient of determination (*R*^2^) and *P*-value for the slope. The steepest slopes and highest R^2^ values are consistently seen for ^232^Th, showing its main role in radiological risk in Farta District soils.

Figure  7 presents a comprehensive set of regression analyses (panels a–l) exploring the relationships between measured activity concentrations of ^226^Ra, ^232^Th and ^40^K and key derived radiological risk indicators. The results reveal important patterns that illuminate the radiological significance of each radionuclide in the study area. In Fig.  7a–c, the correlations between the activity concentrations of ^226^Ra, ^232^Th and ^40^K with the radium equivalent activity (Ra*_eq_*) are shown. As expected, all three radionuclides exhibited strong linear relationships with Ra*_eq_*, with ^232^Th (Fig.  7b) showing the steepest slope, reflecting its greater weighting in the Ra*_eq_* formulation. This highlights the dominant influence of thorium on cumulative radiological hazard indices, as Ra*_eq_* is more sensitive to changes in Th concentrations than to Ra or K. Figure  7d–f illustrates the dependency of the internal hazard index (*H*_in_) on the activity concentrations of ^226^Ra, ^232^Th and ^40^K, respectively. Notably, Hin is most strongly influenced by ^232^Th (Fig.  7e), followed by ^226^Ra (Fig.  7d), with ^40^K (Fig.  7f) contributing marginally. This is consistent with the relative radiotoxicity and inhalation pathways of these radionuclides, where ^232^Th and ^226^Ra have higher alpha-emitting potential and thus contribute more significantly to internal dose. In Fig.  7g–i, the external hazard index (*H*_ex_) is plotted against the radionuclides. Again, ^232^Th (Fig.  7h) shows the most substantial correlation, reinforcing its central role in external radiological exposure. However, ^226^Ra (Fig.  7g) also contributes significantly, while ^40^K (Fig.  7i) exerts only a modest influence due to its lower dose conversion factor. Figure  7j–l presents the annual effective dose equivalent (A*ED*) as a function of radionuclide concentrations. *A*_ED_, being a function of the ADR, exhibits a pattern similar to that observed for *H*_in_ and *H*_ex_. Specifically, *A*_ED_ increases most significantly with ^232^Th levels (Fig.  7k), followed by ^226^Ra (Fig.  7j) and least with ^40^K (Fig.  7l). The regression results confirm that ^232^Th is the primary contributor to the estimated annual dose received by individuals from terrestrial gamma radiation in the study region. Across all panels, the linear regressions are statistically significant (*P* ± 0.001), with moderate to high coefficients of determination (*R*^2^), particularly in associations involving ^232^Th. The presence of 95% confidence intervals around each regression line provides a measure of uncertainty, reflecting natural variability and possible heterogeneity in radionuclide distribution among samples. The *P*-value checks how meaningful the observed linear relationship is. A *P*-value less than the usual significance level, such as *P* < 0.05, suggests that the relationship is significant and probably didn’t happen by random chance. When the *P*-value for a linear regression is >0.05, it is usually not statistically significant at the 5% significance level. In a research setting, this means we do not reject the null hypothesis. The null hypothesis states that there is no linear relationship between the independent variable (the activity concentration of a specific radioisotope) and the dependent variable (a radiological safety parameter). Thus, a *P*-value > 0.05 suggests that the observed correlation might be due to random chance. There isn’t enough statistical evidence to say that the radioisotope reliably predicts that specific safety parameter. The shaded area around the regression line shows the 95% confidence interval for the average predicted value of the dependent variable. A narrow confidence band means the model’s predictions are precise. In contrast, a wider band points to more uncertainty. These plots show a strong, positive linear relationship between the activity concentration of each radioisotope and the calculated Ra_eq_. This close connection makes sense because the Ra_eq_ formula is a direct linear combination of the activity concentrations. The high *R*^2^ values, which are likely near 1.0, and the very low *P*-values will confirm that each isotope significantly contributes to the total Ra_eq_. The slope of each regression line closely matches the internationally accepted conversion factors used in the calculation.

In the end, Fig.  7 collectively underscores the predominant influence of ^232^Th on all radiological hazard indices and dose estimates, followed by ^226^Ra. At the same time, ^40^K—despite often being present in fairly abundant concentrations—plays a somewhat minor role. This pattern results from differences in radiotoxicity, decay characteristics and weighting in radiological risk models. Explicitly, ^232^Th has the greatest dose conversion and hazard coefficients; it gives rise to greater radiotoxic potential because of its decay chain and dominates both external and internal dose pathways. Conversely, ^226^Ra and especially ^40^K are less significant to Ra_eq_, dose rates and effective dose because they have lower radiotoxicities and smaller weighting factors in the formulas for their doses. These are critical findings for environmental radiation risk assessment; it means that mitigation efforts should primarily focus on control zones rich in ^232^Th within this study area.

### Comparative dose–parameter analysis

**Fig. 8:**
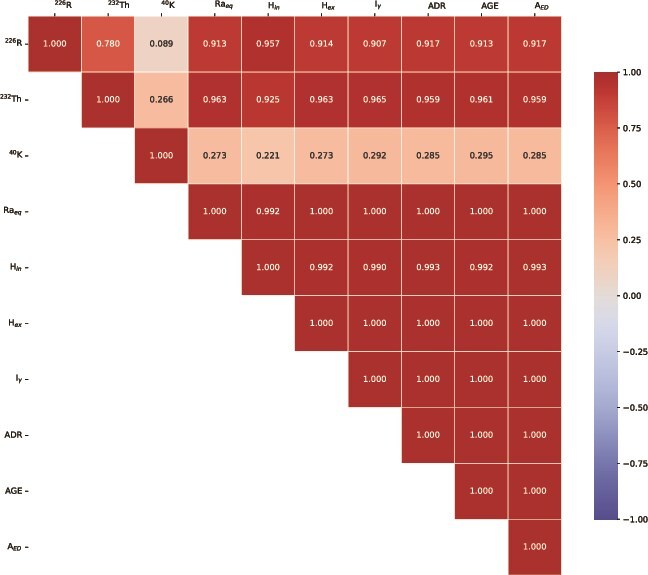
Correlations between radionuclides and radiation risks (Pearson’s).

This dataset includes 27 soil samples analyzed for ^226^Ra (12–342 Bq.kg^−1^), ^232^Th (10–321 Bq.kg^−1^) and ^40^K (65–671 Bq.kg^−1^) activity concentrations. Results reported here indicated strong positive correlations between ^226^Ra and ^232^Th (*r* ≈ 0.92), suggesting common geogenic origins from uranium–thorium minerals; however, ^40^*K* showed much weaker associations with them (*r <* 0.3), implying different potassium-bearing sources (see Fig. 8). Radiological indices calculated included Ra*_eq_* (58–783 Bq.kg^−1^), hazard indices H*_in_*: 0.16–2.11, H*_ex_*: 0.08–1.06 and dose parameters ADR: 28–354 nGy.h^−1^, *A*_ED_: 0.03–0.43 *mSv*.*y*^−1^. Only 14.8% of the samples exceeded the Ra_eq_ recommended safe value of UNSCEAR at 370 Bq.kg^−1^. Correlation coefficients gave almost ideal relationships (*r* ≈ 0.99) between Ra_eq_ and indices of hazards, thus confirming their interdependence in the process of risk assessment. Correlations for moderate ^40^K-dose (*r* ≈ 0.6–0.7) reflected its secondary role in gamma exposure. Mean *A*_ED_(0.17 mSv.y^−1^) remained below the ICRP public limit (1 mSv.y^−1^) [19]. However, localized elevated risks were identified; ELCR indicated up to 0.15 × 10^−2^. This shows that site-specific evaluations are important, especially in such a geologically distinct region where thorium-rich bedrock dominates the radiological profile.

In Fig. 8, the heatmap reveals strong positive correlations (*r >* 0.9, *P <* 0.001) between ^226^Ra and all radiological indices (Ra_eq_, *H*_in_, H_ex_, I*_γ_*, ADR, AGE), reflecting its dominant contribution to radiation hazards due to its high activity concentrations (up to 342 Bq.kg^−1^) and energetic decay products. Similarly, ^232^Th exhibits nearly identical correlation patterns (*r >* 0.9) with these indices, underscoring its comparable role in dose accumulation, particularly through gamma emissions from its decay chain. Both radionuclides show near-perfect alignment with Ra*_eq_* (*r* ≈ 0.99), as the index directly incorporates their weighted activities and with *H*_in_/*H*_ex_ (*r* ≈ 0.98–0.99), confirming their primary influence on internal and external exposure risks. The representative level index (*I_γ_*) also strongly correlates with Ra/Th (*r* ≈ 0.98), as it scales linearly with their concentrations. Dose-rate parameters (ADR, AGE) follow the same trend (*r* ≈ 0.95–0.97), as both isotopes contribute significantly to terrestrial gamma radiation. These results highlight that ^226^Ra and ^232^Th are the principal drivers of radiological hazards in the study area, with their combined effects necessitating careful monitoring in regions exceeding safety thresholds.

**Fig. 9:**
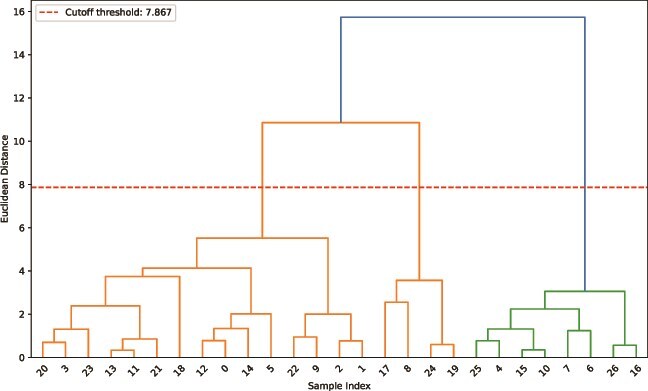
Dendrogram of radionuclides and radiation parameters, Ward’s method, Euclidean distance. Optimal cutoff is at 7.867, *n* = 27.

The hierarchical clustering dendrogram featured in Fig.  9 displays the results of how 27 environmental samples were grouped based on their radioactive multicriteria evaluation, including radiological hazard indices. These samples incorporate the radionuclide concentrations of ^226^Ra, ^232^Th and ^40^K, as well as secondary parameters like Ra_eq_, ADR, *H*_ex_, *H*_in_, *A*_ED_ and ELCR. All values underwent a scaling process to mitigate biases from differing magnitudes. The dendrogram was constructed using Ward’s linkage method with Euclidean distance as the similarity metric, showing the radiological similarity between the samples, or how samples are merged into progressively larger clusters. The red dashed horizontal line denotes the value of cut-off cluster separation, which achieved optimum silhouette score maximization for dissimilarity between clusters while maintaining cohesion within clusters. Analysis at this threshold reveals three clusters that show the samples can be grouped into three radiologically significant categories. These clusters most likely indicate changes in the natural abundance of radioelements, in the associated health hazard parameters and in the context of environmental risk assessment may change the spatial zoning approach of the region. The hierarchical structure also reveals how certain samples with similar radiological profiles group together at lower linkage distances, underscoring the robustness of their shared characteristics.

**Fig. 10:**
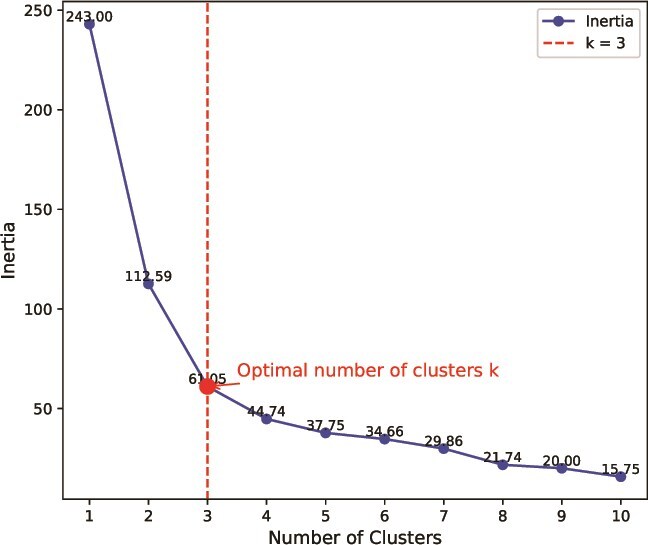
Elbow plot of *K*-means inertia for *k* = 1 to 10 applied to standardized variables (^226^Ra, ^232^Th, ^40^K, Ra_eq_, ADR, *H*_in_, *H*_ex_, A_ED_, ELCR). The elbow at *k* = 3, marked by a red dashed line, indicates the best balance between cohesion and simplicity.

The elbow method evaluates the consistency between the results of hierarchical agglomerative clustering and *K*-means clustering through the cluster structure’s schemata. The Elbow Method plot also finds the best number of clusters for grouping the measurements of radionuclides and their radiological parameters (Ra_eq_, ADR, *H*_ex_, *H*_in_, *A*_ED_, ELCR). In Fig. 10, the *x*-axis shows the number of clusters (*k*), while the *y*-axis indicates how well samples fit within a cluster, known as inertia. Lower values of inertia indicate better cluster cohesion. As *k* ranges from 1 to 10, inertia drops sharply at first, then levels off after a certain point, forming an ‘elbow’. The red dashed line marks the optimal number of clusters. Beyond this point, adding more clusters does not significantly improve the model fit. This indicates that the dataset naturally divides into three groups, representing low, medium and high radioactivity profiles or other unexplored patterns in radiological hazard parameters. The inertia values at *k* = 3 confirm that after the elbow point, adding more clusters yields smaller benefits. This validates choosing *k* = 3 for further analysis (Fig. 10).

Identifying these clusters (low, medium and high radioactivity profiles) through cluster analysis has important practical uses for environmental management and public health. These clusters can be turned into zoning maps that show areas with similar radiological risk profiles. Such maps would be valuable for:

- Targeted monitoring: Focusing monitoring efforts and resources on areas with medium or high radioactivity profiles, leading to more efficient data collection.
- Risk management and mitigation: Supporting land-use planning, agricultural practices and potential cleanup strategies in areas with high radiological hazards, thus reducing public exposure.
- Public awareness and policy-making: Providing clear information to local authorities and residents, helping them create tailored public health advisories and regulations based on the specific radiological characteristics of each zone. This approach goes beyond simple data reporting and offers a way to protect the environment and manage health proactively.

### Health data integration

Recent evidence shows that the cancer burden in Ethiopia has been steadily increasing over the past decade. The Global Cancer Observatory (GLOBOCAN) and national analyses indicate rising incidence, mortality and disability-adjusted life years (DALYs), largely driven by population growth, aging and lifestyle changes [40, 41]. Breast cancer has become the most common cancer among Ethiopian women, with registry data from Addis Ababa and hospital studies reporting more cases and later-stage presentations [42, 43]. Cervical cancer remains a leading cause of cancer death, strongly linked to a high prevalence of human papillomavirus (HPV), especially genotypes HPV-16 and -18 [44, 45].

In addition, the risk of hepatocellular carcinoma is high due to widespread exposure to aflatoxin, often combined with chronic hepatitis B virus (HBV) infection [46, 47]. Environmental and occupational exposures also play a significant role. Unsafe pesticide use and air pollution are worsened by localized risks from elevated natural background radiation, particularly from terrestrial radon gas in certain geological formations and high-energy gamma radiation from volcanic bedrock. These factors can contribute to overall cancer risk through chronic, low-dose exposure [1, 2, 10–13, 48–50].

Together, these findings emphasize the urgent need to strengthen cancer registries, expand HPV vaccination and screening programs, implement aflatoxin mitigation strategies and establish public health initiatives. These initiatives should focus on mapping high-radon areas and promoting radon-resistant construction, along with stricter regulation of other environmental carcinogens to tackle the growing cancer threat in the country.

## CONCLUSIONS

This study gives the first solid baseline look at natural radionuclides, specifically ^226^Ra, ^232^Th and ^40^K, in the agricultural soils of Farta District, Ethiopia. We used HPGe gamma-ray spectroscopy for the measurements, and the results paint an interesting picture. On average, the soils’ radium equivalent activity (Ra_eq_) lands right around 370 Bq/kg, which matches the international screening guideline. Still, a few spots went over this level, so those areas need a closer look. It’s worth clarifying how these reference values work. The international numbers used here: 370 Bq/kg for Ra_eq_, 1 mSv/y for effective dose and hazard indices at or below 1 were meant for managing exposures from artificial radiation or practices using NORM. They don’t apply to unmodified natural backgrounds. For this study, we just used them as a kind of yardstick to flag areas with higher radionuclide levels than the world average. We’re not saying these places break any regulatory rules. The same goes for the ADR and AED: both averages fall below the ICRP’s public exposure limit of 1 mSv/y; other model-based indicators—including the ELCR, AGDE and gamma representative index—consistently exceeded global reference values in several locations. Critically, these indices are screening tools derived from established conversion factors, not direct evidence of adverse health outcomes. Their exceedance does not imply clinical risk but rather identifies priority areas for more granular investigation. Among the 27 samples analyzed, only 37% exhibited an internal hazard index (*H*_in_) below the safety threshold of 1, and 44% fell below the external hazard index (*H*_ex_) threshold, underscoring the need for site-specific attention rather than district-wide generalizations. Statistical analyses revealed strong positive correlations between ^226^Ra, ^232^Th and all radiological hazard parameters, with ^232^Th emerging as the dominant contributor to dose estimates. Hierarchical clustering further resolved the dataset into three distinct radiological profiles: low, medium and high, providing an evidence-based framework for targeted monitoring and intervention.

This study is subject to several limitations that must be acknowledged. First, the relatively small sample size (*n* = 27) constrains the generalizability of findings across the entire district. Second, the absence of direct radon (^222^Rn) measurements, a critical pathway for internal dose, represents a significant gap. Third, the scope was deliberately limited to three primordial radionuclides; anthropogenic markers (e.g. ^137^Cs, ^60^Co) were not assessed. Fourth, without concurrent epidemiological or clinical data from the region, health risk inferences remain model-based and speculative.

Radon surveys, both indoor and outdoor, are urgently needed to quantify inhalation doses, particularly in areas with elevated ^226^Ra. Food-chain transfer studies should assess radionuclide uptake in staple crops (e.g. rice, onions) to evaluate dietary exposure pathways. Seasonal sampling is warranted to capture temporal variability driven by irrigation, fertilizer application and hydrological processes. Spatial zoning maps, informed by the cluster analysis, should be developed to guide land-use planning and prioritize high-risk areas for remediation or longitudinal monitoring. Anthropogenic radionuclide screening would complement this baseline by identifying potential contamination from industrial or agricultural sources.

In summary, this study establishes a reproducible radiological baseline for the Farta District, a geologically distinct region influenced by the East African Rift system. The data confirm that while mean activity concentrations of ^226^Ra and ^232^Th exceed global averages, they remain within ranges observed in other high-background areas worldwide. The identification of localized hotspots through clustering provides a spatially explicit framework for allocating monitoring resources efficiently.

Crucially, by explicitly distinguishing between model-based screening metrics and empirical health evidence, this work avoids overinterpretation while still delivering actionable insights for environmental managers and policymakers. The integration of this baseline with future health registry data—should such data become available—would ultimately enable validation of dose–response relationships and strengthen evidence-based radiation protection standards in Ethiopia.

## Data Availability

The data and source code supporting this study will be provided upon reasonable request to the corresponding author.
